# Transformative Approaches in Breast Cancer Detection: Integrating Transformers into Computer-Aided Diagnosis for Histopathological Classification

**DOI:** 10.3390/bioengineering12030212

**Published:** 2025-02-20

**Authors:** Majed Alwateer, Amna Bamaqa, Mohamed Farsi, Mansourah Aljohani, Mohamed Shehata, Mostafa A. Elhosseini

**Affiliations:** 1 Department of Computer Science, College of Computer Science and Engineering, Taibah University, Yanbu 46421, Saudi Arabia; mwateer@taibahu.edu.sa (M.A.); mjohni@taibahu.edu.sa (M.A.); 2 Department of Computer Science and Informatics, Applied College, Taibah University, Madinah 41461, Saudi Arabia; abamaqa@taibahu.edu.sa; 3 Department of Information Systems, College of Computer Science and Engineering, Taibah University, Yanbu 46421, Saudi Arabia; mafarsi@taibahu.edu.sa; 4 Department of Bioengineering, Speed School of Engineering, University of Louisville, Louisville, KY 40292, USA; mohamed.shehata@louisville.edu; 5 Computers and Control Systems Engineering Department, Faculty of Engineering, Mansoura University, Mansoura 35516, Egypt

**Keywords:** breast cancer (BC), deep learning (DL), digital histopathology, transformers

## Abstract

Breast cancer (BC) remains a leading cause of cancer-related mortality among women worldwide, necessitating advancements in diagnostic methodologies to improve early detection and treatment outcomes. This study proposes a novel twin-stream approach for histopathological image classification, utilizing both histopathologically inherited and vision-based features to enhance diagnostic precision. The first stream utilizes Virchow2, a deep learning model designed to extract high-level histopathological features, while the second stream employs Nomic, a vision-based transformer model, to capture spatial and contextual information. The fusion of these streams ensures a comprehensive feature representation, enabling the model to achieve state-of-the-art performance on the BACH dataset. Experimental results demonstrate the superiority of the twin-stream approach, with a mean accuracy of 98.60% and specificity of 99.07%, significantly outperforming single-stream methods and related studies. Statistical analyses, including paired *t*-tests, ANOVA, and correlation studies, confirm the robustness and reliability of the model. The proposed approach not only improves diagnostic accuracy but also offers a scalable and efficient solution for clinical applications, addressing the challenges of resource constraints and increasing diagnostic demands.

## 1. Introduction

Breast cancer (BC) remains a leading cause of cancer-related mortality among women worldwide [1,2]. With over 2.3 million new cases reported in 2020 alone [3], the global burden of BC underscores the urgent need for advancements in diagnostic methodologies to improve early detection and treatment outcomes. Early and accurate diagnosis is critical for reducing mortality and improving patient outcomes [4,5].

Traditional diagnostic approaches, such as clinical examinations, mammography, and histopathological analysis, have significantly contributed to early detection. However, the intricate nature of BC necessitates continual advancements in diagnostic precision. Recent innovations in artificial intelligence (AI) and deep learning (DL) have emerged as transformative tools for analyzing medical data, enabling more accurate and timely diagnoses [6,7,8]. These technologies excel at identifying complex patterns and relationships within datasets, facilitating efficient and personalized patient care [9,10].

Vision transformers (ViTs), originally developed for natural language processing (NLP), have shown remarkable capabilities in medical image analysis [11,12]. Their ability to capture long-range dependencies and contextual relationships makes them particularly valuable for deciphering intricate patterns in histopathology images [13,14].

This study proposes a novel twin-stream approach for BC histopathology classification, integrating both histopathologically inherited features and vision-based data. The first stream utilizes Virchow2, a pretrained model designed to extract high-level histopathological features, while the second stream employs Nomic, a vision-based transformer model, to analyze spatial and contextual information. The fusion of these streams ensures a comprehensive feature representation, enhancing diagnostic accuracy and robustness. Data augmentation techniques, including random rotations, flips, and Gaussian noise injection, are employed to improve generalization.

By proposing this innovative framework, the study seeks to advance computer-aided diagnosis of breast cancer, providing a scalable, efficient, and resource-friendly solution for healthcare providers with the following contributions:

- Twin-Stream Architecture: A novel architecture combining two streams—Virchow2 for histopathological feature extraction and Nomic for vision-based transformer modeling—offering a comprehensive representation of breast cancer histopathological data.
- Integration of ViTs: Demonstrated the application of ViTs for histopathological image analysis, addressing long-range dependencies and contextual relationships often missed by traditional methods.
- State-of-the-Art Performance: Achieved a mean accuracy of 98.60% and specificity of 99.07% on the BACH dataset, surpassing the performance of existing single-stream and hybrid models.
- Comprehensive Statistical Validation: Performed rigorous statistical analyses, including paired *t*-tests, ANOVA, and correlation studies, to confirm the robustness and reliability of the proposed approach.
- Benchmark Comparisons: Demonstrated superior performance against benchmark models like Inception-v3, ResNet50, ADSVM with RANet, and Google Teachable Machine CNN, hig ighting the advantages of the twin-stream methodology.
- Class-Wise Evaluation: Provided detailed insights into class-specific performance, showing improved detection for categories like invasive, benign, normal, and in situ classes, even in challenging scenarios.
- Scalable and Efficient Framework: Designed a computationally efficient solution that can be adapted to resource-constrained environments, addressing the increasing demand for scalable diagnostic tools in clinical settings.
- Clinical Applicability: Hig ighted the potential for the proposed twin-stream approach to be implemented in real-world healthcare scenarios, offering enhanced diagnostic precision and consistency.

The remainder of this paper is organized as follows: Section 2 reviews related works, hig ighting the research gap addressed by this study. Section 3 describes the dataset used. Section 4 details the proposed methodology. Section 5 presents experimental configurations and results. Section 6 discusses the results and their medical relevance. Section 7 concludes with future research directions.

## 2. Related Studies

Breast cancer (BC) remains one of the most widespread malignancies affecting women globally, necessitating innovative approaches to improve diagnostic performance and expedite treatment. Recent advancements in computational techniques, particularly in histopathological image analysis (HIA), have significantly enhanced classification and detection methodologies [15,16,17,18]. This section reviews key methodologies, hig ighting their contributions and identifying gaps that justify the exploration of ViTs in BC diagnosis.

Golatkar et al. [19] developed a deep learning (DL) approach using a fine-tuned Inception-v3 CNN to classify hematoxylin and eosin (H&E)-stained breast tissue slides into four classes: normal, benign, in situ carcinoma, and invasive carcinoma. By extracting patches based on nuclear density to eliminate uninformative areas, they achieved an average accuracy of 85% and 93% for distinguishing non-cancerous from malignant cases. Building on this, Zhou et al. [20] introduced a two-component method that combined anomaly detection with a support vector machine (ADSVM) and a resolution-adaptive network (RANet). Their approach achieved peak accuracies of 97.75% (multiclass) and 99.25% (binary) on the BACH 2018 dataset, outperforming ResNet and DenseNet while reducing computational time by 50%. Similarly, Vesal et al. [21] proposed a transfer learning-based approach using Inception-V3 and ResNet50 for classifying breast histology images. After addressing color variations through normalization, Inception-V3 achieved a slightly superior accuracy of 97.08%, compared to ResNet50’s 96.66%. In another study, Vizcarra et al. [22] combined shallow (SVM) and deep (CNN) learners for BC histology classification. While individual models achieved accuracies of 79% and 81%, respectively, their fusion improved accuracy to 92%, hig ighting the potential of hybrid approaches. Finally, Kone et al. [23] developed a hierarchical CNN system for classifying histology images into four pathologies, achieving 99% accuracy on the BACH benchmark and 96% on an extension dataset, demonstrating its effectiveness in automated pathology classification. Collectively, these studies underscore the progress made in BC histopathology classification, yet they also reveal gaps in fully leveraging complementary information from histopathological data.

### Research Gap

Despite notable advancements, existing methods often fail to fully exploit complementary information in histopathological data. Single-stream architectures typically focus on either high-level morphological patterns or low-level visual details, but not both. Additionally, the integration of ViTs for histopathological image analysis remains underexplored, despite their proven effectiveness in capturing long-range dependencies and contextual relationships. The proposed twin-stream approach addresses this gap by combining histopathologically inherited features from Virchow2 and vision-based features from Nomic. This dual-stream architecture, coupled with advanced fusion techniques and dynamic feature selection, offers a robust, accurate, and scalable solution for BC diagnosis, significantly improving accuracy, specificity, and consistency.

## 3. Materials

Study Design and Ethical Considerations: This study focuses on the automatic classification of histology images, emphasizing the importance of early BC detection to improve patient outcomes. BC is a significant health concern worldwide, especially for women, necessitating advanced diagnostic tools. This work employs a dataset of H&E-stained breast tissue images to address the complexities of HIA. The dataset contains both microscopy and whole-slide images (WSIs), labeled pixel-wise into four classes, serving as a key resource for developing automated BC detection and diagnosis systems.

Patient Selection and Characteristics: The dataset comprises carefully curated H&E-stained breast tissue images, classified into four histological categories: normal, benign, in situ carcinoma, and invasive carcinoma. Annotation was performed by two experienced medical professionals to ensure accuracy, with images displaying discrepancies in labeling omitted from the dataset. In total, there are 400 microscopy images, with 100 images representing each histological category. While patient demographic details are partially available, they are limited in scope.

Imaging Techniques: The imaging dataset includes both microscopy images and WSIs, each with unique specifications:

- Microscopy Images: Dimensions of 2048 × 1536 pixels, with a pixel scale of 0.42 μm × 0.42 μm, and individual image file sizes ranging from 10 to 20 MB.
- Whole-Slide Images: Stored in .svs format, these images have varying sizes, for example, 42,113 × 62,625 pixels, a pixel scale of 0.467 μm/pixel, and memory requirements of approximately 8 GB when stored as a numpy array or 200–250 MB in .svs format.

Data Annotation and Analysis: Annotations for both microscopy and WSIs were conducted by two medical experts, with any disagreements leading to the exclusion of specific images. The labeled WSIs include coordinates that enclose regions categorized as benign, in situ carcinoma, and invasive carcinoma, with these coordinates stored in XML files for precise mapping and classification.

Data Categorization: The dataset is structured to facilitate the development of classification models:

- Microscopy Images: Classified into 4 categories: (a) normal, (b) benign, (c) in situ carcinoma, and (d) invasive carcinoma.
- Whole-Slide Images: Annotated with regions labeled by coordinates for benign, in situ carcinoma, and invasive carcinoma areas, aligning with the microscopy image classes for consistency.

**Figure 1:**
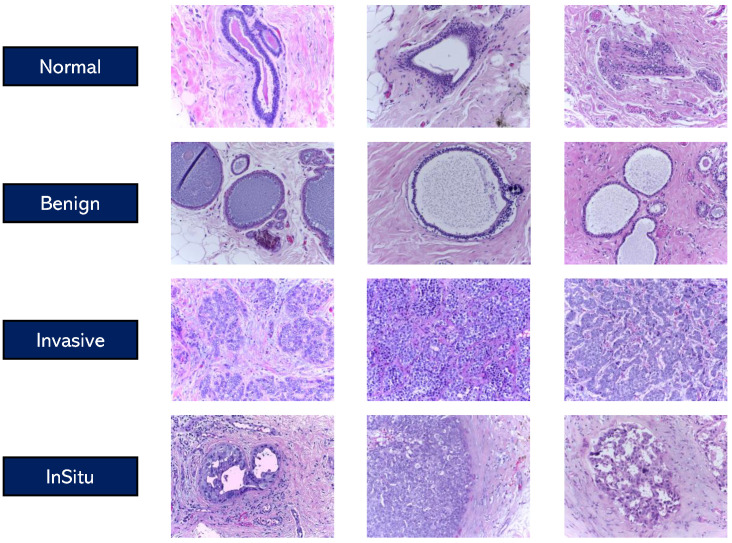
Visualization of pre-extracted patches from the four histological classes (i.e., Normal, Benign, Invasive, and InSitu) from the BACH dataset.

Examples of the categorized image samples are displayed in Figure 1, illustrating the distinctions across the four classes of breast tissue histology.

Data Availability: This dataset, part of the BACH challenge, is available for public research use. Researchers can access the dataset, including the latest comprehensive version for the BACH challenge, at https://iciar2018-challenge.grand-challenge.org (Accessed on 12 December 2024 ).

## 4. Methodology

**Figure 2:**
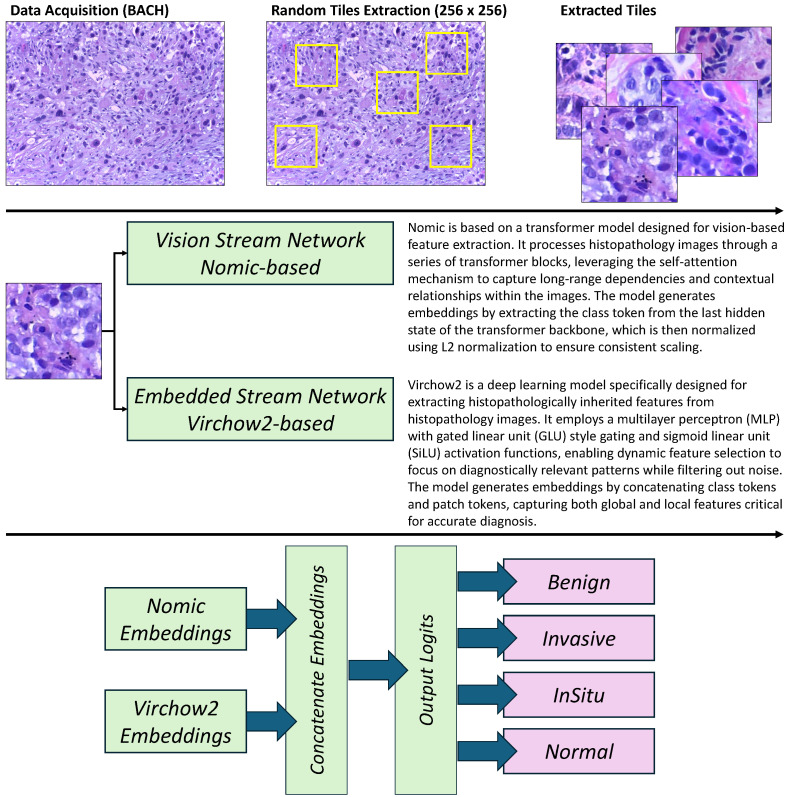
Overview of the twin-stream framework for BC histopathology classification. The framework integrates histopathologically inherited features from Virchow2 and vision-based features from Nomic, followed by feature fusion and classification to achieve accurate and robust diagnosis.

Breast cancer (BC) represents a significant public health challenge, underscoring the need for accurate and improved diagnostic techniques and methodologies. The integration of histopathological imaging with ML methodologies offers a promising avenue to enhance diagnostic precision. Therefore, the proposed methodology (Figure 2) employs a twin-stream approach to extract complementary features from histopathology images, utilizing both histopathologically inherited and vision-based data. This twin-stream design is motivated by the need to capture both high-level morphological patterns and low-level visual details, which are essential for accurate diagnosis.

The first stream uses Virchow2, a pretrained deep learning model specifically designed to extract high-level histopathologically inherited features from histopathology images. The second stream employs Nomic, a vision-based feature extraction model, to analyze spatial and contextual information from the same histopathology images. The fusion of these two streams ensures a comprehensive feature representation, enhancing the model’s diagnostic capabilities.

### 4.1. Feature Extraction: Histopathologically Inherited Features Using Virchow2

The first stream utilizes Virchow2, a deep learning model inspired by the principles of histopathology, which involve analyzing tissue structures at a microscopic level to identify abnormalities [24,25]. Virchow2 processes input images through a series of transformations and convolutional layers, generating embeddings that capture intricate patterns and morphological characteristics of breast cancer tissue [26]. These embeddings are particularly effective at identifying features such as cell nuclei, tissue architecture, and other histopathological markers indicative of malignancy.

Virchow2 incorporates a multilayer perceptron (MLP) with gated linear unit (GLU) style gating and sigmoid linear unit (SiLU) activation functions. The GLU mechanism allows for dynamic feature selection, enabling the model to focus on the most relevant patterns in the histopathological data while ignoring irrelevant or noisy information. This dynamic gating mechanism is particularly useful in medical imaging, where the presence of artifacts or variations in image quality can complicate feature extraction [24,25].

The embeddings are derived from both class tokens c and patch tokens P, which are concatenated to form a comprehensive feature representation as shown in Equation (1), where $c\in R^{d}$ is the class token, $P_{i}\in R^{d}$ represents the *i*-th patch token, *N* is the number of patches, and ⊕ denotes concatenation. This ensures that the model captures both global and local features critical for accurate diagnosis.

(1) $$E_{\mathrm{Virchow}2}=c⊕\left(\frac{1}{N}\times \sum_{i=1}^{N}P_{i}\right)$$

The GLU mechanism is mathematically expressed as in Equation (2), where σ is the sigmoid activation function, ⊙ denotes element-wise multiplication, and $W_{1},W_{2}$ and $b_{1},b_{2}$ are learnable weights and biases, respectively. This mechanism enables the model to dynamically filter out irrelevant features, enhancing its ability to focus on diagnostically relevant patterns.

(2) $$\mathrm{GLU}\left(x\right)=\sigma \left(W_{1}x+b_{1}\right)⊙\left(W_{2}x+b_{2}\right)$$

### 4.2. Feature Extraction: Vision-Based Features Using Nomic

The second stream employs Nomic, a vision-based feature extraction model, to analyze spatial and contextual information from the same histopathology images. Nomic is based on a transformer architecture, which has proven hig y effective in capturing long-range dependencies and contextual relationships in image data [27]. The model processes the images through a series of transformer blocks, extracting embeddings $E_{\mathrm{Nomic}}\in R^{d}$ that encode visual patterns such as texture, color, and structural irregularities.

These features are particularly useful for identifying visual cues such as nuclear pleomorphism, glandular formation, and stromal characteristics, which are key indicators of malignancy in breast cancer. The Nomic model uses a Nomic processor to preprocess the input images, followed by a transformer backbone that generates a last hidden state. The class token from this hidden state is extracted and normalized via L2 normalization to ensure consistent scaling of the feature vectors. By combining these embeddings with the histopathologically inherited features from Virchow2, the model achieves a more holistic understanding of the tumor characteristics, enhancing its diagnostic capabilities.

The transformer architecture in Nomic is based on the self-attention mechanism, which computes attention scores between all pairs of tokens in the input sequence. The attention scores are computed as in Equation (3), where Q, K, and V are the query, key, and value matrices, respectively, and $d_{k}$ is the dimensionality of the keys. This mechanism allows the model to capture long-range dependencies and contextual relationships in the input images, which are critical for accurate feature extraction.

(3) $$\mathrm{Attention}\left(Q,K,V\right)=\mathrm{SoftMax}\left(\frac{Q\times K^{T}}{\sqrt{d_{k}}}\right)\times V$$

### 4.3. Feature Fusion: Integrating Histopathological and Vision-Based Features

To ensure a robust and comprehensive feature representation, the embeddings extracted from Virchow2 and Nomic are integrated through a fusion mechanism. This fusion process combines the strengths of histopathologically inherited and vision-based data while minimizing redundancy. The embeddings from both streams are normalized using L2 normalization, as shown in Equation (5), and concatenated to form the fused embedding $E_{\mathrm{fused}}$, as expressed in Equation (4).

(4) $$E_{\mathrm{fused}}=\mathrm{Norm}\left(E_{\mathrm{Virchow}2}\right)⊕\mathrm{Norm}\left(E_{\mathrm{Nomic}}\right)$$

(5) $$\mathrm{Norm}\left(x\right)=\frac{x}{{∥x∥}_{2}}$$

This fusion process is followed by a series of fully connected (FC) dense layers, which further refine the combined feature representation. Each dense block consists of an FC layer, a LeakyReLU activation function, and a dropout layer to prevent overfitting. The integration of these features is critical for capturing the complex interplay between morphological and visual characteristics, enabling the model to make more informed decisions during classification. The use of dropout layers ensures that the model generalizes well to unseen data, reducing the risk of overfitting and improving its robustness in real-world scenarios.

Additionally, to further enhance the fusion process, we employ a multi-scale attention mechanism that weighs the importance of different feature channels before concatenation. This mechanism assigns higher weights to channels that contribute more significantly to the final classification decision, ensuring that the most discriminative features are prioritized. The multi-scale attention mechanism is formulated as in Equation (6) where $W_{a}$ and $b_{a}$ are learnable parameters, and [⋯, ⋯] denotes channel-wise concatenation. The attention weights $A_{\mathrm{scale}}$ are then used to scale the feature maps before fusion, ensuring that only the most informative features are retained.

(6) $$A_{\mathrm{scale}}=\mathrm{SoftMax}\left(W_{a}\left[E_{\mathrm{Virchow}2},E_{\mathrm{Nomic}}\right]+b_{a}\right)$$

The LeakyReLU activation function is defined as in Equation (7), where α is a small positive constant (typically 0.01). This activation function helps mitigate the “dying ReLU” problem, where neurons can become inactive and stop learning.

(7) $$\mathrm{LeakyReLU}\left(x\right)=\left\{\begin{array}{cc} x & \mathrm{if}\;x>0,\\ \alpha \times x & \mathrm{otherwise} \end{array}\right.$$

The overall algorithm is presented in Algorithm 1.
**Algorithm 1:** The twin-stream feature extraction and fusion process for breast cancer histopathology classification.  1Function **TwinStreamFeatureExtractionAndFusion**(I)// Input: Histopathology image *I*. Output: Fused feature representation $E_{\mathrm{fused}}$.(  2// **Step 1: Preprocessing**  3Normalize input image *I* to ensure consistent scaling.  4// **Step 2: Feature Extraction with Virchow2**  5Pass *I* through Virchow2 to extract high-level histopathological features: Compute class token *c* and patch tokens $P_{i}$ (i=1,⋯,N). Concatenate class and patch tokens to form $E_{\mathrm{Virchow2}}$:$$E_{\mathrm{Virchow2}}=c⊕\frac{1}{N}\sum_{i=1}^{N}P_{i}$$Apply Gated Linear Unit (GLU) mechanism for dynamic feature selection:$$\mathrm{GLU}\left(x\right)=\sigma \left(W_{1}x+b_{1}\right)⊙\left(W_{2}x+b_{2}\right)$$
  6// **Step 3: Feature Extraction with Nomic**  7Pass *I* through Nomic to extract vision-based features. Compute attention scores using self-attention mechanism:$$\mathrm{Attention}\left(Q,K,V\right)=\mathrm{SoftMax}\left(\frac{Q\times K^{T}}{\sqrt{d_{k}}}\right)\times V$$Extract embeddings $E_{\mathrm{Nomic}}$ from the last hidden state.  8// **Step 4: Feature Fusion**  9Normalize embeddings from both streams:$$\mathrm{Norm}\left(x\right)=\frac{x}{{∥x∥}_{2}}$$10Concatenate normalized embeddings:$$E_{\mathrm{fused}}=\mathrm{Norm}\left(E_{\mathrm{Virchow}2}\right)⊕\mathrm{Norm}\left(E_{\mathrm{Nomic}}\right)$$11Apply multi-scale attention to weigh feature channels:$$A_{\mathrm{scale}}=\mathrm{SoftMax}\left(W_{a}\left[E_{\mathrm{Virchow}2},E_{\mathrm{Nomic}}\right]+b_{a}\right)$$12Scale feature maps using $A_{\mathrm{scale}}$ before fusion.13// **Step 5: Classification**14Pass $E_{\mathrm{fused}}$ through fully connected layers with LeakyReLU activation:$$\mathrm{LeakyReLU}\left(x\right)=\left\{\begin{array}{cc} x & \mathrm{if}\;x>0,\\ \alpha \times x & \mathrm{otherwise} \end{array}\right.$$Output final classification decision.15Return $E_{\mathrm{fused}}$.16End Function

### 4.4. Data Augmentation During Learning

To enhance the model’s generalization capabilities and robustness, data augmentation techniques are employed during the training process. These techniques include random rotations, flips, color jittering, and Gaussian noise injection, which simulate variations in histopathology images that may occur due to differences in staining, lighting, and tissue preparation. Data augmentation ensures that the model is exposed to a diverse range of image variations, improving its ability to handle real-world scenarios where such variations are common.

Mathematically, data augmentation can be represented as a transformation function T(x), where x is the input image. For example, random rotation can be expressed as in Equation (8), where R(θ) is the rotation matrix for an angle θ.

(8) $$T_{\mathrm{rotate}}\left(x,\theta \right)=R\left(\theta \right)x$$

Similarly, Gaussian noise injection can be expressed as in Equation (9), where n is noise sampled from a Gaussian distribution with zero mean and variance $\sigma^{2}$.

(9) $$T_{\mathrm{noise}}\left(x\right)=x+n,\;n∼N\left(0,\sigma^{2}\right)$$

### 4.5. Performance Metrics

Evaluating a classification model’s performance is mandatory to assess and understand its effectiveness, especially in the task at hand. Key metrics such as accuracy, recall, specificity, precision, F1 score, intersection over union (IoU), balanced accuracy (BAC), Youden’s index, Matthews correlation coefficient (MCC), and Yule’s Q collectively offer a multi-dimensional view of the model’s strengths and weaknesses [28,29]. These metrics capture various performance aspects that can ensure a comprehensive evaluation for breast cancer (BC) classification in histology images.

## 5. Experiments

The experimental setup was designed to evaluate the performance and reliability of the proposed twin-stream model in classifying histopathology images. Data augmentation techniques, including random flipping and cropping, were applied to enhance generalization. The dataset was split into 80% for training and 20% for testing. Evaluation metrics were computed with adjustments for numerical stability [30,31]. Experiments were conducted on a system running Windows 11, equipped with 256 GB RAM and an 8 GB NVIDIA GPU. The implementation was performed in Python using PyTorch 2.6, Timm, and Transformers libraries. Table 1 summarizes the experimental configurations.

**Figure 3:**
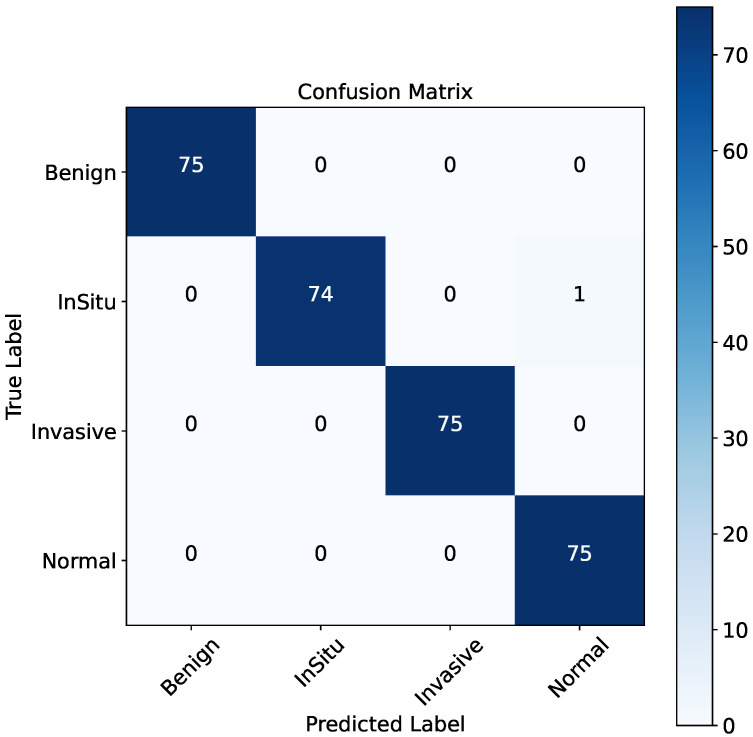
Visualization of the confusion matrix of the four histological classes (i.e., Normal, Benign, Invasive, and InSitu) from the BACH dataset utilizing the two-stream mechanism.

The results for the twin-stream approach are presented in Table 2. Across 15 runs, the model achieved a mean accuracy of 98.60% and specificity of 99.07%, with low standard deviations (e.g., 0.0015 for accuracy) and confidence intervals (e.g., 0.0008 for accuracy). These metrics demonstrate the robustness and stability of the twin-stream architecture in handling histopathological data. Moreover, Figure 3 visualizes the confusion matrix of the four histological classes from the BACH dataset utilizing the two-stream mechanism.

In contrast, Table 3 shows the results for the single-stream approach ($E_{\mathrm{Virchow2}}$). The mean accuracy dropped to 90.97%, and specificity decreased to 93.98%. Higher standard deviations (e.g., 0.0070 for accuracy) and wider confidence intervals (e.g., 0.0036 for accuracy) indicate greater variability. These results hig ight the importance of the complementary information provided by the twin-stream architecture.

### 5.1. Statistical Analysis

#### 5.1.1. Paired *t*-Test and Wilcoxon Signed-Rank Test

The paired *t*-test (T=41.19, p<0.0001) and Wilcoxon signed-rank test (Statistic=0.0, p<0.0001) both indicate that the twin-stream approach significantly outperforms the single-stream method. The paired *t*-test assumes normality of differences, while the Wilcoxon test is non-parametric and does not rely on this assumption. Both tests reject the null hypothesis, with mean accuracies of ($\mu_{TS}=0.9860$) for the twin-stream and ($\mu_{SS}=0.9097$) for the single-stream approach.

#### 5.1.2. One-Way ANOVA and Kruskal-Wallis Test

The one-way ANOVA (F=115413.66, p<0.0001) and Kruskal–Wallis test (H=42.30, p<0.0001) confirm significant performance differences between the twin-stream approach and related studies. ANOVA assumes normally distributed data and equal variances, whereas the Kruskal–Wallis test relaxes these assumptions. Both tests reject the null hypothesis, hig ighting the substantial improvement achieved by the twin-stream architecture.

#### 5.1.3. Effect Size Calculation

The effect size, calculated using Cohen’s *d* (d=14.98), indicates an exceptionally large difference between the twin-stream and single-stream approaches. This underscores the practical significance of the twin-stream model in clinical settings requiring accurate histopathological classification.

#### 5.1.4. Correlation Analysis

Correlation analysis reveals a strong positive relationship between accuracy and specificity for the twin-stream approach. Pearson’s correlation coefficient (r=0.999, p<0.0001) and Spearman’s rho (ρ=1.0, p=0.0) confirm this relationship. The high correlation suggests that the model minimizes false positives while maintaining reliability, even in the presence of potential non-linearities.

#### 5.1.5. Discussion of Combined Results

The statistical analyses provide robust evidence for the superiority of the twin-stream approach. Paired *t*-tests and Wilcoxon signed-rank tests confirm significant performance differences under different assumptions, while confidence interval analysis hig ights the consistency of the method. One-way ANOVA and Kruskal–Wallis tests demonstrate that the twin-stream approach surpasses related studies, with a large effect size (Cohen’s d=14.98) quantifying the magnitude of improvement. Finally, correlation analysis reinforces the robustness of the twin-stream architecture by showing a strong relationship between accuracy and specificity.

### 5.2. Comparison with Related Studies

Table 4 compares the proposed twin-stream approach with existing methods. Golatkar et al. [19] achieved an average accuracy of 85%, Vesal et al. [21] reported 97.08% using Inception-V3, Zhou et al. [20] achieved 97.75%, and Kone et al. [23] reported 0.99 on the BACH dataset. With a mean accuracy of 98.60%, the twin-stream approach not only surpasses these benchmarks but also demonstrates superior consistency, as evidenced by low standard deviations and confidence intervals. This improvement stems from the architecture’s ability to leverage complementary features, enhancing discriminative power and generalization capability.

### 5.3. Comparison with Google Teachable Machine (CNN)

The evaluation results of the proposed model indicate a total of 62 true positives across all classes, yielding an overall accuracy of 71.26%. A class-wise analysis reveals varying levels of performance among the categories. The class labeled “Invasive” demonstrates an impressive accuracy of 85.06%, accompanied by balanced precision and recall values of 0.84 and 0.86, respectively, indicating effective detection of this category.

Conversely, the class “Benign” exhibits lower precision (0.53) and recall (0.59), achieving an accuracy of 81.61%, suggesting challenges in distinguishing benign cases from others. The “Normal” class achieves high accuracy at 90.8%, with balanced precision and recall both reported at 0.73, illustrating the model’s efficacy in correctly identifying non-cancerous cases. Meanwhile, the “InSitu” class shows an accuracy of 85.06% but lower precision (0.44) and recall (0.33), indicating that the model may misclassify some in situ cases.

Therefore, the twin-stream approach proposed in this study represents a significant advancement in histopathological image classification. Its ability to achieve high accuracy, specificity, and consistency across multiple runs underscores its potential for clinical applications. The comparative analysis with related studies further validates its superiority, positioning it as a state-of-the-art method for breast cancer histopathology classification using the BACH dataset.

## 6. Overall Discussion and Medical Relevance

The proposed twin-stream approach marks a significant advancement in histopathological image classification for BC diagnosis. By combining histopathologically inherited features from Virchow2 and vision-based features from Nomic, the model captures both high-level morphological patterns and low-level visual details, addressing the limitations of single-stream methods. Advanced techniques like dynamic feature selection, data augmentation, and robust normalization further enhance its accuracy and reliability.

Experimental results demonstrate the twin-stream model’s superiority, achieving a mean accuracy of 98.60% and specificity of 99.07%, significantly outperforming the single-stream approach (accuracy: 90.97%, specificity: 93.98%). Low standard deviations and confidence intervals hig ight its consistency, while statistical analyses (paired *t*-tests, ANOVA, etc.) confirm its significant improvement, with a large effect size (Cohen’s d=14.98).

The medical relevance is profound. Accurate BC diagnosis is critical for improving patient outcomes, and this model automates histopathology image classification, reducing reliance on manual interpretation, which is time-consuming and error-prone. Its high specificity minimizes false positives, reducing unnecessary interventions, while its ability to distinguish BC subtypes supports personalized treatment planning.

By integrating AI techniques such as ViTs, the twin-stream approach enhances diagnostic accuracy and efficiency, offering a scalable solution for large-scale screening programs. This is especially important in global health, where early detection is key to reducing BC mortality.

With that said, the twin-stream model represents a state-of-the-art solution for BC histopathology classification, with high accuracy, specificity, and consistency. Its scalability and efficiency address resource constraints, making it a valuable tool for clinicians and pathologists.

### 6.1. Computational Efficiency

Computational efficiency is critical for clinical adoption, especially in resource-limited settings. To address this, we conducted an inference time analysis and explored potential optimizations:

- Inference Time: On our experimental setup (Windows 11, 8 GB NVIDIA GPU), the model processes a single 256 × 256 patch in approximately 0.03 s, making it suitable for real-time applications. However, deploying the model on lower-end hardware may require additional optimizations.
- Optimization Strategies:
  – Pruning: Remove redundant neurons and layers without significantly affecting performance.
  – Quantization: Convert the model to lower precision (e.g., FP16 or INT8) to reduce memory usage and accelerate inference.
  – Cloud-Based Deployment: Host the model on cloud platforms to enable remote access and reduce the computational burden on local devices.
  – Edge Computing: Deploy lightweight versions of the model on edge devices for faster inference in clinical settings. These optimizations can enhance the model’s usability in diverse healthcare environments, ensuring scalability and accessibility.

### 6.2. Clinical Adoption and Implications

The integration of AI models into pathology workflows marks a significant advancement in diagnostic efficiency and accuracy. AI-driven tools, such as our twin-stream approach, complement human expertise by flagging suspicious cases, providing second opinions, or prioritizing urgent cases. This reduces the cognitive load on pathologists, enhancing diagnostic precision while maintaining clinical oversight. However, successful adoption requires addressing regulatory approval, clinician education, and trust-building. To facilitate this, we propose standardized guidelines for AI use, training programs for clinicians, and feedback loops between developers and users to refine model performance.

AI-driven histopathological analysis also supports personalized oncology by offering detailed insights into tumor characteristics, enabling precise classification of breast cancer subtypes. This is critical for tailoring treatments, such as distinguishing between in situ and invasive carcinoma to inform interventions like surgery or chemotherapy. Studies [32,33] hig ight how AI models enhance cancer diagnosis and support personalized treatment pathways, underscoring the potential of our twin-stream model to advance precision medicine.

Clinician interaction with AI predictions is another key aspect of adoption. While statistical validation is essential, understanding how pathologists perceive AI tools provides a holistic view of their role in clinical workflows. Transparency, interpretability, and real-world validation are crucial for building trust [32,33]. AI models serve as decision-support tools, offering objective analyses that reduce workload and improve accuracy. High diagnostic accuracy minimizes false positives and negatives, improving patient outcomes and enabling large-scale screening programs. Bridging technical advancements with clinical applicability ensures AI innovations deliver meaningful benefits to healthcare systems and patients.

### 6.3. Dataset Limitations and Suggestions

The BACH dataset, while a valuable benchmark, primarily represents a specific population and staining protocol, which may introduce biases related to demographic distribution, geographic variability, and image acquisition conditions [32,33]. Specifically, we note the following:

- The lack of complete patient-wise origin information in the BACH dataset may limit the generalizability of the model to broader populations.
- Variations in staining protocols, scanner differences, and image quality across institutions could impact the model’s performance when applied to external datasets.

To mitigate these biases, we offer the following recommendations for future studies:

- Incorporate more diverse datasets that include samples from different geographic regions, ethnic groups, and healthcare systems.
- Use domain adaptation techniques to enhance the model’s robustness to variations in staining protocols and imaging conditions.
- Validate the model on external datasets to assess its generalizability and real-world applicability.

These steps will ensure that the model remains reliable and equitable across diverse clinical settings.

### 6.4. Overall Limitations

The proposed CAD framework for BC classification, while innovative, faces several limitations. High computational demands during training may restrict accessibility, and the BACH dataset’s lack of complete patient-wise data could limit generalizability. Applying the framework to other medical imaging tasks requires further validation. Challenges also remain in model interpretability and ethical handling of patient data. Addressing these issues through advancements in computational tools, improved datasets, better interpretability methods, and robust ethical practices will be critical for enhancing the framework’s applicability in medical image analysis.

## 7. Conclusions and Future Work

The twin-stream approach proposed in this study represents a significant advancement in the field of histopathological image classification for breast cancer diagnosis. By utilizing complementary features from both histopathologically inherited and vision-based data, the model achieves state-of-the-art performance, as evidenced by its high accuracy (98.60%), specificity (99.07%), and consistency across multiple runs. The fusion of Virchow2 and Nomic embeddings ensures a comprehensive feature representation, capturing both high-level morphological patterns and low-level visual details critical for accurate diagnosis. Statistical analyses, including paired *t*-tests, ANOVA, and correlation studies, further validate the superiority of the twin-stream approach over single-stream methods and related studies. The model’s robustness, demonstrated by low standard deviations and confidence intervals, hig ights its reliability and potential for real-world clinical applications.

Future work will focus on enhancing the model’s performance and applicability through several key areas. First, integrating additional data modalities, such as genomic or proteomic data, could improve diagnostic precision by providing a more comprehensive understanding of tumor characteristics. Second, advanced fusion techniques, like attention-based mechanisms or graph neural networks, may refine feature representation and boost classification accuracy. Third, extending the model to handle multi-modal imaging data, such as combining histopathology with radiology images, could enhance diagnostic capabilities across imaging domains. Finally, large-scale clinical validation studies will be crucial to evaluate the model’s generalizability and effectiveness in diverse settings.

## Figures and Tables

**Table 1:** Experimental configurations and specifications.

Configuration Aspect	Details
Learning Rate	$1\times 10^{-4}$
Tile Size	256×256
Epochs	50
Batch Size	16
Data Augmentation Techniques	Random flipping and cropping
Train-Test Split	80% training, 20% testing
Evaluation Metrics	Accuracy, Recall, IoU, Specificity, Precision, and F1
Metric Stability	Small constant added for numerical stability

**Table 2:** Representation of the performance metrics (e.g., accuracy and recall) for the suggested approach of the twin-stream using the BACH dataset (with the pre-extracted patches).

Runs	Precision	Recall	F1	Accuracy	Specificity
Mean	0.9728	0.9720	0.9724	0.9860	0.9907
Std	0.0029	0.0030	0.0030	0.0015	0.0010
CI	±0.0015	±0.0015	±0.0015	±0.0008	±0.0005

**Table 3:** Representation of the performance metrics (e.g., accuracy and recall) after depending only on a single stream ($E_{\mathrm{Virchow2}}$).

Runs	Precision	Recall	F1	Accuracy	Specificity
Mean	0.8533	0.8193	0.8360	0.9097	0.9398
Std	0.0115	0.0141	0.0126	0.0070	0.0047
CI	±0.0058	±0.0071	±0.0064	±0.0036	±0.0024

**Table 4:** Tabular comparison of the related studies that utilized the BACH histopathology dataset and the current study.

Study	Approach	Results
Golatkar et al. [19]	DL-based method using fine-tuned Inception-v3 CNN for classifying H&E-stained breast tissue images.	Average accuracy of 85% across all classes. Significant improvement over previous benchmark with 93% accuracy for non-cancer vs. malignant.
Zhou et al. [20]	Innovative BC diagnosis approach integrating anomaly detection (ADSVM) and resolution adaptive network (RANet).	Achieved top accuracies of 97.75% for multiclass classification and 99.25% for binary classification at the image level. Marked improvements in both classification accuracy and computational efficiency over ResNet and DenseNet, with a 50% reduction in computational time.
Vesal et al. [21]	Transfer learning-based breast histology image classification using Inception-V3 and ResNet50 CNNs. Addressed color variations with normalization.	Inception-V3 achieved an average test accuracy of 97.08%, outperforming ResNet50 (96.66%). Transfer learning-based approach demonstrated effectiveness in histology image classification.
Vizcarra et al. [22]	Image classification pipeline for BC diagnosis, integrating shallow (SVM) and deep (CNN) learners.	Integrated system achieved the highest accuracy of 92%, surpassing individual learners. Fusion algorithms demonstrated potential for enhancing clinical design support in BC diagnosis.
Kone et al. [23]	Hierarchical CNN system for automated BC pathology classification using microscopic histology image analysis.	Achieved remarkable accuracy of 0.99 on the test split for the BACH dataset and 0.96 for the extension dataset. Automated hierarchical CNN system demonstrated efficacy in accurately classifying BC pathologies.
Current Study	A twin-stream approach	Accuracy of 98.60% on the testing subset (See Table 2).

## Data Availability

The data presented in this study are openly available at https://iciar2018-challenge.grand-challenge.org [BACH’s dataset] [https://iciar2018-challenge.grand-challenge.org/] [2018].
